# Metabolic engineering of terpene metabolism in lavender

**DOI:** 10.1186/s43088-024-00524-7

**Published:** 2024-07-08

**Authors:** Ojo Michael Oseni, Reza Sajaditabar, Soheil S. Mahmoud

**Affiliations:** Department of Biology, The University of British Columbia, Okanagan Campus, 3333 University Way, Kelowna, BC V1V 1V7 Canada

**Keywords:** Lavender, Isoprenoids, Metabolic engineering, Monoterpenes, Terpene synthase

## Abstract

**Background:**

Several members of the Lamiaceae family of plants produce large amounts of essential oil [EO] that find extensive applications in the food, cosmetics, personal hygiene, and alternative medicine industries. There is interest in enhancing EO metabolism in these plants.

**Main body:**

Lavender produces a valuable EO that is highly enriched in monoterpenes, the C_10_ class of the isoprenoids or terpenoids. In recent years, substantial effort has been made by researchers to study terpene metabolism and enhance lavender EO through plant biotechnology. This paper reviews recent advances related to the cloning of lavender monoterpene biosynthetic genes and metabolic engineering attempts aimed at improving the production of lavender monoterpenes in plants and microbes.

**Conclusion:**

Metabolic engineering has led to the improvement of EO quality and yield in several plants, including lavender. Furthermore, several biologically active EO constituents have been produced in microorganisms.

## Background

Lavender (genus *Lavandula*) belongs to the family Lamiaceae and is known for producing large amounts of essential oil [EO]. The genus *Lavandula* encompasses over 30 known species, each characterized by a unique EO profile [1, 2]. The presence of specific monoterpenes in lavender EO contributes to its value, making it a significant resource in the production of perfumes, medicinal products, food flavorings, and antiseptics [3, 4]. The most abundant monoterpenes in lavender EO are linalool, linalyl acetate, borneol, camphor, and 1,8-cineole [5, 6]. Other notable monoterpenes include limonene, lavandulol, lavandulyl acetate, and α-terpineol [4, 7, 8]. In addition, lavender produces certain monoterpenes in response to environmental conditions [8, 9].

Among all lavender species, *Lavandula angustifolia*, *Lavandula latifolia*, and their natural hybrid, *Lavandula x intermedia*, hold significant commercial importance [10]. *L.* *angustifolia* is highly valued in the perfumery industry due to its high levels of linalool and linalyl acetate. EO obtained from *L. latifolia* is important in the medical sector because of its high concentrations of linalool, camphor, and 1,8-cineole. *L.* *x intermedia* EO contains constituents found in both parents, and is widely used in personal care and hygiene products, [3].

This review highlights recent efforts aimed at biotechnological improvement of lavender EO in engineered plants. It also provides information on the production of lavender EO constituents in microorganisms.

## Main text

### Methodology

Published articles were sourced from various search engines and databases, including Web of Science, Google Scholar, ScienceDirect, Scopus, and SciFinder. Keywords used in the search included lavender, along with essential oil, glandular trichomes, gene cloning, transformation, metabolism, engineering, terpene synthases, terpene biosynthesis, and genome editing. Additionally, separate searches were conducted for yeast, bacteria, and cyanobacteria in conjunction with key monoterpenes from lavender EO such as linalool, 1,8-cineole, and borneol.

### Terpenes

The terpenoids (terpenes), also known as isoprenoids, represent the most abundant class of plant secondary metabolites. They serve crucial roles in plant growth, development, overall metabolism, and defense against predators, diseases, and competition [11]. Many terpenes have applications in cosmetics, pharmaceuticals, insecticides, and potential biofuels. In plants, the biosynthesis of terpenes is divided into three stages. The first stage involves the production of the common precursors, isopentenyl diphosphate [IPP] and its isomer dimethylallyl diphosphate [DMAPP] via the 2-C-methyl-D-erythritol 4-phosphate [MEP] pathway, also known as the 1-deoxy-D-xylulose-5-phosphate [DXP] pathway, and/or mevalonate [MVA] pathway. During the second stage, intermediates such as geranyl diphosphate [GPP], farnesyl diphosphate [FPP], and geranylgeranyl diphosphate [GGPP] are synthesized from IPP/DMAPP by isoprenyl diphosphate synthases [IDSs], also known as prenyltransferases. The final stage involves the production of various terpenes, catalyzed by terpene synthases [TPSs] such as linalool synthase [LINS] and 1,8-cineole synthase [CINS], along with terpene-modifying enzymes. It has been shown that the enzymes involved in terpene production have distinct subcellular localizations: all MEP pathway enzymes are located in plastids, while MVA pathway enzymes are found in the cytosol or peroxisomes [13, 14]. IDSs and TPSs exhibit more diverse localizations and are frequently associated with the subcellular site of terpene biosynthesis [12].

### Glandular trichomes [GTs]

GTs are specialized plant structures dedicated to the synthesis and accumulation of EO. Lavender possesses two types of GTs: peltate GTs and capitate GTs. Peltate GTs produce and store a significant amount of monoterpene-rich EO in lavender [6, 15]. Each peltate GT comprises a basal cell, anchoring it to the epidermis, a stalk cell, up to eight secretory cells, and a storage cavity.[16]. A plasma membrane separates the storage cavity from the secretory cells [17, 18]. Within the secretory cells, EO synthesis occurs in two compartments: the cytosol and the leucoplast, where the MVA and MEP pathways operate, respectively [17, 19, 20]. EO constituents synthesized in the plastids via the MEP pathway are transported to the cytosol and subsequently secreted into the storage cavity, either directly or after processing [18].

Trichome formation, which is widely studied in Arabidopsis, is regulated in part by transcription factors [TFs] that can have either positive or negative effects. Additionally, other TFs function upstream or downstream of these regulators [21]. Recently, Zhang et al. [22] studied GT formation in lavender using a genomics approach, identifying several TFs belonging to R2R3-MYB subfamily associated with GT development.

### Monoterpene biosynthesis

**Fig. 1:**
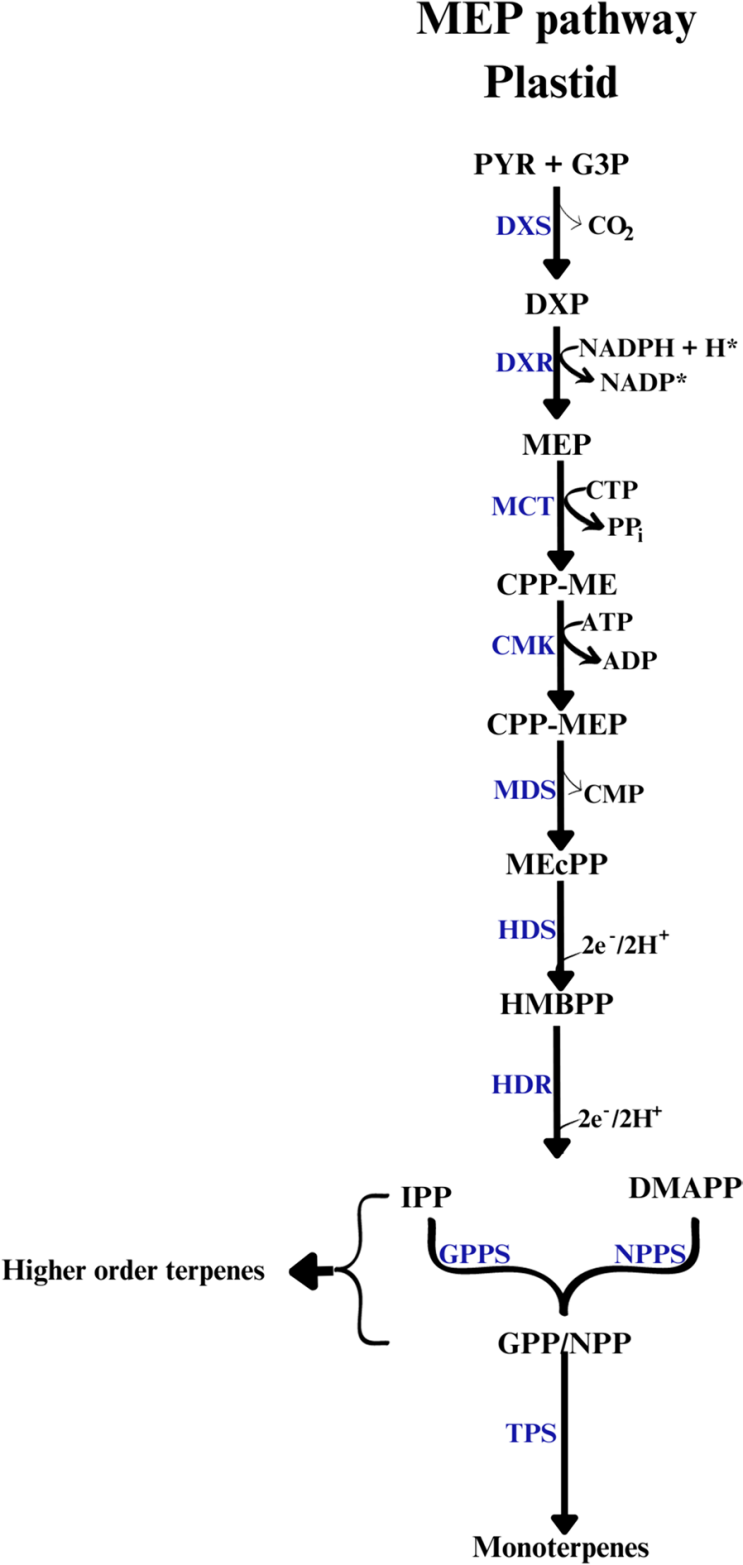
Biosynthesis of isopentenyl diphosphate [IPP] and dimethylallyl diphosphate [DMAPP] through 2-C-methyl-D-erythritol 4-phosphate [MEP] pathway. PYR [pyruvate], G3P [glyceraldehyde 3–phosphate], DXP [1-deoxy-D-xylulose-5-phosphate], CPP-ME [cytidyl diphosphate-methyl-D-erythritol], CPP-MEP [cytidyl diphosphate-methyl-D-erythritol 4-phosphate], MEcPP [2-C-methyl-D-erythritol -2,4-cyclodiphosphate], HMBPP [1-hydroxy-2-methyl-2-(E)-butenyl-4-diphosphate], GPP [geranyl diphosphate], NPP [neryl diphosphate], and GGPP [geranylgeranyl diphosphate]

Monoterpenes are synthesized within the plastids of photosynthetic organisms, utilizing primary metabolites [12, 23]. In plants, chloroplasts serve as the major sites for monoterpene biosynthesis via the MEP pathway (Fig. 1) [24]. The pathway initiates with the condensation and decarboxylation of pyruvate [PYR] and glyceraldehyde 3-phosphate [G3P] by DXP synthase [DXS], yielding DXP. Subsequently, DXP reductoisomerase [DXR] catalyzes the reduction of DXP to MEP via NADPH-dependent isomerization [25]. MEP undergoes a series of enzymatic reactions involving MEP cytidyltransferase [MCT], 4-(cytidine 5ˈ-diphospho)- 2-C-methyl-D-erythritol-kinase [CMK], and 2-C-methyl-D-erythritol-2,4-cyclodiphosphate [MEcPP] synthase [MDS], leading to the formation of MEcPP [26, 27]. MEcPP is further reduced to 4-hydroxy-3-methylbut-2-enyl diphosphate [HMBPP] by HMBPP synthase [HDS]. IPP and DMAPP, the final products of MEP pathway, are generated in a ratio of approximately 5:1through the reduction of HMBPP by HMBPP reductase [HDR] [28–30]. Prenyltransferases, including geranyl diphosphate synthase [GPPS] and neryl diphosphate synthase [NPPS], catalyze the condensation of IPP and DMAPP to synthesize GPP and neryl diphosphate [NPP], which are precursors of monoterpenes [31]. In addition to monoterpenes, IPP/DMAPP derived from the MEP pathway is used to produce numerous other terpenes including photosynthetic pigments such as phytol and carotenoid precursors [32, 33].

### Monoterpene metabolism in lavender

**Fig. 2:**
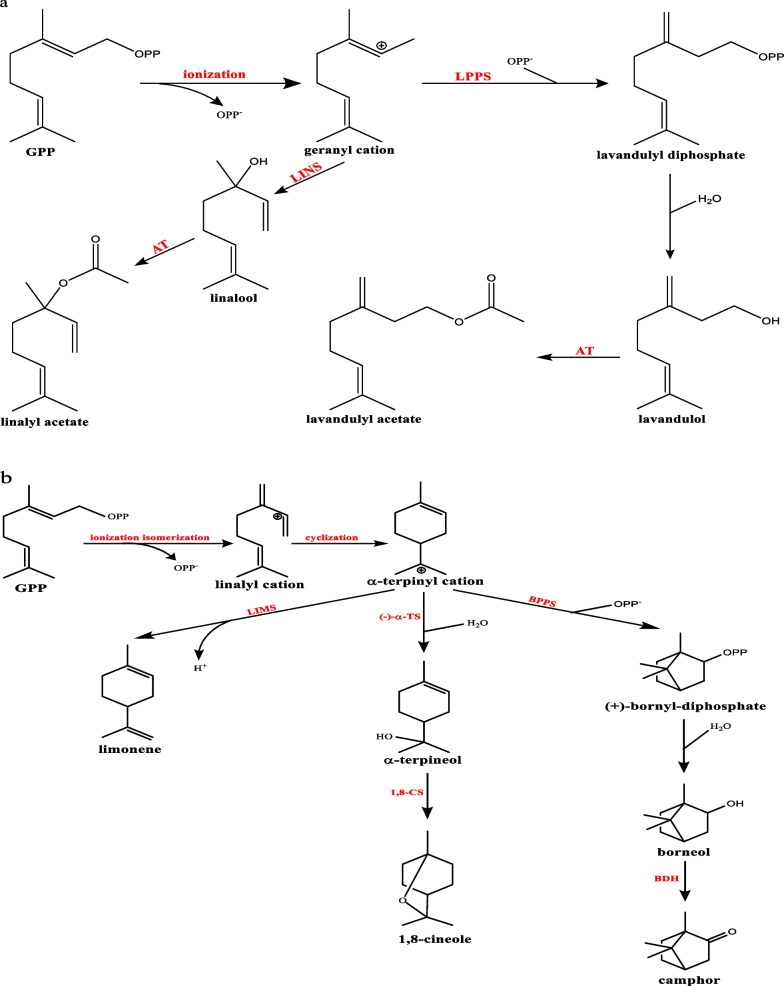
Metabolism of acyclic **a** and cyclic **b** monoterpenes in lavender. LIMS [limonene synthase], (−)-α-TS [(−)-α-terpineol synthase], 1,8 CS [1,8-cineole synthase], BPPS [bornyl diphosphate synthase], BDH [borneol dehydrogenase], LINS [linalool synthase], AT [acetyltransferase], and LPPS [lavandulyl diphosphate synthase]

In line with other plant species, TPSs are responsible for catalyzing the conversion of the GPP substrate into cyclic and acyclic monoterpenes within the secretory cells of lavender GTs [34, 35]. Numerous researchers have undertaken efforts to clone TPS genes and characterize their functionality in vitro, aiming to elucidate their roles in EO production (Table 1). For instance, Landmann et al. [1] employed a homology-based PCR approach to clone two monoterpene synthases [monoTPSs]: limonene synthase [*LaLIMS*] and LINS [*LaLINS*], and one sesquiterpene synthase [sesquiTPSs]: bergamotene synthase [*LaBERS*] from *L. angustifolia* leaves and flowers. Subsequently, the cloned cDNAs were expressed in *Escherichia coli. LaLIMS* produced limonene, terpinolene, camphene, α-pinene, β-myrcene, and traces of β-phellandrene, while *LaLINS* solely produced (R)-(−)-linalool, and *La*BERS converted FPP into bergamotene. In addition, Demissie et al. [36] reported the cloning and functional characterization of β-phellandrene synthase [*La*β*PHLS*] in *E. coli*. Their results showed that the recombinant *LabPHLS* converted GPP and NPP into β-phellandrene. In 2012, Demissie et al. [4] identified the CINS [*LiCINS*] gene from *L. x intermedia* and cloned it into *E. coli*. The resulting bacterially generated recombinant protein, approximately 63 kDa in size, converted GPP mainly into 1,8-cineole. As reported again by Demissie et al. [37], *L. x intermedia* lavandulyl diphosphate synthase [*LiLPPS*] was cloned using a homology-based cloning method and expressed in *E. coli*. The resulting protein, with a molecular weight of about 34.5 kDa, catalyzed the fusion of two DMAPP units to form lavandulyl diphosphate [LPP] in vitro*.* The works of Demissie et al. [4, 36, 37] were continued by the research of Sarker et al. [2, 7, 38], who cloned and expressed borneol dehydrogenase [*LiBDH*], caryophyllene synthase [*LiCPS*], and two alcohol acetyltransferases [*LiAAT-3 and LiAAT-4*] genes in *E. coli*. The recombinant *LiBDH* protein converted borneol into camphor, the recombinant *LiCPS* protein converted FPP into 9-epicaryophyllene, and both recombinant *LiAAT*-3 and *LiAAT*-4 proteins converted lavandulol to lavandulyl acetate. In 2015, Benabdelkader et al. [9] cloned two monoTPSs and one sesquiTPS from *L. pedunculata* and functionally identified them as fenchol synthase [*LpFENS*], α-pinene synthase [*LpPINS*], and germacrene A synthase [*LpGEAS*]. Interestingly, while the expression patterns of *FENS* and *PINS* genes aligned with the enzyme product accumulation profile, this correlation was not observed for *GEAS*. Next, Adal et al. [8] identified and characterized a monoTPS gene, 3-carene synthase [*Li3CARS*], from *L. x intermedia*. The results showed that the recombinant Li3CARS transformed GPP into 3-carene as the major product (Fig. 2). In 2019, the same researcher reported the cloning of *S*-LINS [*LiS*-LINS] gene from *L. x intermedia* in bacteria. The cloned *LiS-LINS* catalyzed the conversion of GPP into S-linalool as the sole product [39]. In 2023, Adal et al. [40] cloned lavender (+)- bornyl diphosphate synthase [*LiBPPS*] in bacteria, and the recombinant *LiBPPS* promoted the conversion of GPP to (+)- bornyl diphosphate [BPP] as the main product, accompanied by the formation of several minor monoterpene compounds. In a recent study, Ling et al. [41] identified terpene synthase 7 [*LaTPS7*] and terpene synthase 8 [*LaTPS8*] genes from *L. angustifolia* during the budding phases. Subsequently, they cloned these TPSs into *E. coli* and *Nicotiana benthamiana*. The recombinant *LaTPS7* generated nine different products in vitro, including camphene, myrcene, and limonene, while *LaTPS8* produced eight volatiles using GPP and NPP as substrate. Moreover, the overexpression of *LaTPS7* in *N. benthamiana* resulted in the synthesis of limonene, whereas *LaTPS8* yielded α-pinene and sylvestrene.

**Table 1:** Cloning and metabolic engineering of lavender genes

Study type	Gene studied	Source of gene	Species	Performance	References
Gene cloning	Limonene synthase [*LaLIMS*]	*L. angustifolia*	*E. coli*	*LaLIMS* catalyzed the formation of (R)-(+)-limonene, terpinolene, (1R,5S)-(+)-camphene, (1R,5R)-(+)-α-pinene, β-myrcene and traces of α-phellandrene	[1]
Gene cloning	Linalool synthase [*LaLINS*]	*L. angustifolia*	*E. coli*	*LaLINS* produced exclusively (R)-(−)-linalool	[1]
Gene cloning	Bergamotene synthase [*LaBERS*]	*L. angustifolia*	*E. coli*	*La*BERS transformed farnesyl diphosphate to bergamotene	[1]
Gene cloning	β-phellandrene synthase [*LaβPHLS*]	*L. angustifolia*	*E. coli*	The recombinant *La*βPHLS did not utilize farnesyl diphosphate as a substrate, it converted geranyl diphosphate and neryl diphosphate into β-phellandrene as the major product	[36]
Gene cloning	1,8-Cineole synthases [*Li*CINS]	*L.* × *intermedia*	*E. coli*	The bacterially produced protein converted geranyl diphosphate to 1,8-cineole	[4]
Gene cloning	Lavandulyl diphosphate synthase [*LiLPPS*]	*L.* × *intermedia*	*E. coli*	The bacterially produced protein specifically catalyzed the head-to-middle condensation of two dimethylallyl diphosphate units to lavandulyl diphosphate	[37]
Gene cloning	Borneol dehydrogenase [*LiBDH*]	*L.* × *intermedia*	*E. coli*	The bacterially produced enzyme converted borneol to camphor	[2]
Gene cloning	9-epicaryophyllene synthase [*LiCPS*]	*L.* × *intermedia*	*E. coli*	The recombinant protein converted farnesyl diphosphate to 9-epicaryophyllene. Also, few monoterpenes were produced when assayed with geranyl diphosphate	[7]
Gene cloning	Acetyltransferases [*LiAAT*]	*L.* × *intermedia*	*E. coli*	*LiAAT-4* has a better catalytic efficiency than *LiAAT-3*, with lavandulol serving as the preferred substrate for both enzymes	[38]
Gene cloning	Fenchol synthase [*Lp*FENS]	*L. pedunculata*	*L. pedunculata*	Expression of *LpFENS* gene matched the accumulation profile of the enzyme products	[9]
Gene cloning	*α*-pinene synthase [*Lp*PINS]	*L. pedunculata*	*L. pedunculata*	Expression profiles of *LpPINS* gene matched the accumulation profile of the enzyme products	[9]
Gene cloning	Germacrene A synthase [*Lp*GEAS]	*L. pedunculata*	*L. pedunculata*	Expression profiles of *LpGEAS* gene does not match the accumulation profile of the enzyme products	[9]
Gene cloning	*S*-linalool synthase [*LiS*-LINS]	*L.* × *intermedia*	*E. coli*	Recombinant *LiS-LINS* catalyzed the conversion of the universal monoterpene precursor geranyl diphosphate to *S*-linalool as the sole product	[39]
Gene cloning	3-Carene synthase [*Li3CARS*]	*L.* × *intermedia*	*E. coli*	The recombinant *Li3CARS* converted geranyl diphosphate into 3-carene as the major product	[8]
Gene cloning & Metabolic engineering	(+)‑Bornyl diphosphate synthase [(+)*-LiBPPS*]	*L.* × *intermedia*	*E. coli*	The recombinant (+)*-LiBPPS* catalyzed the conversion of geranyl diphosphate to bornyl diphosphate as a major product, and a few other minor products of monoterpenes	[40]
Metabolic engineering	Terpene synthases [*LaTPS7*]	*L. angustifolia*	*E. coli*	The in vitro studies revealed that LaTPS7 generated nine distinct compounds, including camphene, myrcene, and limonene	[41]
Metabolic engineering	Terpene synthases [*LaTPS8*]	*L. angustifolia*	*E. coli*	*LaTPS8* enzymatically generated eight volatiles by utilizing geranyl diphosphate and nerolidyl diphosphate as substrates	[41]
Development Engineering	Short vegetative phase [*LaSVP*]	*L. angustifolia*	*A. thaliana*	The expression of the *LaSVP* in *A. thaliana* delayed flowering and affected flower organs. Also, two of the highest expressing lines produced sepals instead of petals and failed to develop proper seed pods as they were sterile	[43]
Development Engineering	*LaAGAMOUS-like* [*LaAG-*like]	*L. angustifolia*	*A. thaliana*	The results revealed that all transgenic plants bloomed earlier than wild-type controls	[44]
Development Engineering	*LaSEPALLATA3-like* [*LaSEP3-*like]	*L. angustifolia*	*A. thaliana*	The results revealed that all transgenic plants bloomed earlier than wild-type controls	[44]
Metabolic engineering	Stress-responsive transcription factor [*LaMYC4*]	*L. angustifolia*	*A. thaliana* / *N. benthamiana*	*LaMYC4* overexpression increased the levels of sesquiterpenoids, including caryophyllene	[45]
Metabolic engineering	1-Deoxy-D-xylulose-5-phosphate synthase [*DXS*]	*A. thaliana*	*L. latifolia*	Transgenic plants accumulated significantly more essential oils	[46]
Metabolic engineering	*Agrobacterium rhizogenes* genes	*A. rhizogenes*	*L.* × *intermedia*	Plants were transformed with wild-type *A. rhizogenes.* Most regenerated plants showed dwarfism. Only nine of the 45 regenerated plants formed flower buds. Many transgenics showed a significantly lower productivity of essential oil. The relative percentage of linalool and linalyl acetate decreased in most of the regenerated plants	[47]

### Cloning other lavender genes

As shown in Table 1, other aspects of lavender have also been studied. For instance, Guitton et al. [42] conducted a study on the concentration of volatile organic compound [VOC] in *L. angustifolia* throughout inflorescence growth. They found that calyces were the primary sources of VOC accumulation, with three major VOC groups dominating the global fragrance bouquet of inflorescences. The transition of VOCs occurred between the opening of the inflorescence's first flower and the beginning of seed set. There is a need to develop more knowledge on the molecular features of bloom initiation and development in lavender. Wells et al. [43] studied the short vegetative phase [*LaSVP*] gene of *L. angustifolia* and transformed it into *A. thaliana*. Their results showed that expression of *LaSVP* in *A. thaliana* delayed flowering, resulted in the production of sepals instead of petals, and prevented the formation of seed pods. Additionally, Adal et al. [44] used RNA-Seq and transcript profiling to identify several TFs potentially regulating floral development in lavender. Their study focused on the roles of two TFs, *LaAGAMOUS-like* [*LaAG-like*] and *LaSEPALLATA3-like* [*LaSEP3-like*], in flower development. *LaAG-like* and *LaSEP3-like* cDNAs were overexpressed in Arabidopsis plants. The results revealed that all transgenic plants exhibited earlier flowering compared to wild-type controls. Furthermore, mildly overexpressed plants grew normally, but those that excessively expressed the transgene had curling leaves. Another researcher, Dong et al. [45] focused on a bHLH TF, *LaMYC4*, an important regulator for plant terpenoid biosynthesis. This gene was isolated from *L. angustifolia* following methyl jasmonate [MeJA] treatment and overexpressed in Arabidopsis and tobacco. Results showed that overexpression of *LaMYC4* enhanced sesquiterpenoids, including caryophyllene.

### Metabolic engineering to produce lavender monoterpenes

#### Engineering lavender plants

The initial lavender metabolic engineering endeavor to improve the plastidial MEP pathway for the synthesis of the precursors IPP and DMAPP, was conducted by Munoz-Bertomeu et al. [46]. In their study, Munoz-Bertomeu et al. up-regulated DXS, an enzyme catalyzing the initial step in the MEP pathway. They expressed a cDNA encoding the *A. thaliana* DXS into spike lavender. Gas chromatography/mass spectrometry [GC–MS] analyses indicated that transgenic plants produced significantly higher EOs compared to control plants, with the accumulated EOs maintained in the T_1_ generation. In another effort, Tsuro and Ikedo [47] infected calli derived from lavandin (*L.* × *intermedia*) leaves with *Agrobacterium rhizogenes*, and regenerated plants. However, the regenerated plantlets displayed dwarfism due to short internodes, and low EO content. Moreover, Adal et al. [40] expressed the (+)-*LiBPPS* gene in both sense and antisense orientation. They observed that when (+)-*LiBPPS* was orientated in the antisense orientation, there was a reduction in the synthesis of (+)-borneol and camphor, while plant growth and development remained unaffected. Conversely, plants with the sense-orientated (+)-*LiBPPS* produced higher levels of borneol and camphor, but their growth and development were adversely affected.

#### Engineering microbes to produce lavender EO constituents

Researchers are also considering engineering microorganisms (Table 2) for the (eventual) large-scale production of lavender EO monoterpene constituents. The following are prominent examples of such studies:

**Table 2:** Microbial production of major monoterpenes present in lavender

Monoterpene	Gene/Source of gene	Host Species	Strategy	Concentration	References
Linalool	Linalool synthase/*Actinidia arguta*	*Saccharomyces cerevisiae*	Introducing a fusion protein comprised of linalool synthase and farnesyl diphosphate synthase enzymes with a polypeptide linker between them	240.64 ± 5.31 µg/L	[48]
Linalool	Linalool synthase/ *Actinidia arguta*	*Saccharomyces cerevisiae*	Introducing isopentenol utilization pathway, N-terminal truncated linalool synthase, and a double-mutated farnesyl diphosphate synthase. Medium optimization	142.88 mg/L	[49]
Linalool	Linalool synthase/*Mentha citrata*	*Saccharomyces cerevisiae*	Introducing linalool synthase alongside mevalonate pathway genes. Applying double mutation in linalool synthase and farnesyl diphosphate synthase	53.14 mg/L	[50]
Linalool	Linalool synthase/ *Cinnamomum osmophloeum*	*Saccharomyces cerevisiae*	Dual metabolic engineering of mevalonate pathway in mitochondria and cytoplasm. Medium optimization. Applying batch fermentation	23.45 mg/L	[51]
Linalool	Linalool synthase/ *Mentha citrata*	*Saccharomyces cerevisiae*	Integration of an inducible sensor array in genomic DNA, and applying the sensor molecules: xylose, anhydrotetracycline, vanillic acid, and IPTG to induce the expression of linalool synthase	9.9 ± 0.3 µg/L	[52]
Linalool	Linalool synthase/*Streptomyces clavuligerus*	*Escherichia coli*	Applying a synthetic protein scaffold containing 4 domains for linalool synthase, 1 domain for isopentenyl diphosphate, and 1 domain for geranyl diphosphate synthase. Medium and temperature optimization. Applying fed-batch fermentation	1523.2 mg/L	[56]
Linalool	Geranyl diphosphate synthase/*Abies grandis*	*Escherichia coli*	Introducing geranyl diphosphate synthase and mevalonate pathway genes. Overexpression of isopentenyl diphosphate isomerase	63 ± 5.6 mg/L	[57]
Linalool	Linalool synthase/*Streptomyces clavuligerus*	*Escherichia coli*	Modification of linalool synthase ribosomal binding site, and adding a fusion tag to enhance linalool synthase solubility. Introducing a geranyl diphosphate synthase from *A. grandis*. Using a bioreactor with fed-batch fermentation	1027.3 mg/L	[58]
Linalool	Linalool synthase/ *Actinidia arguta*	*Synechosystis* sp	Introducing a codon-optimized linalool synthase from *A. arguta*	11.6 mg/L	[62]
Linalool/1,8-Cineole	Linalool synthase and 1,8-Cineole synthase/*streptomyces clavuligerus*	*Escherichia coli*	Introducing linalool synthase, 1,8-cineole synthase, mevalonate pathway genes, and an N-terminal truncated geranyl diphosphate synthase	363.3 ± 57.9 mg/L linalool/ 116.8 ± 36.4 mg/L 1,8-cineole	[60]
1,8-Cineole	1,8-Cineole synthase/*Streptomyces clavuligerus*	*Synechococcus elongatus*	Photosynthetic production of 1,8-cineole by introducing a codon-optimized 1,8-cineole synthase from *Streptomyces clavuligerus*	105.6 µg/g wet cell	[63]
1,8-Cineole	1,8-Cineole synthase/*Hypoxilon sp*	*Rhodotorula toruloides*	Introducing an N-terminal truncated geranyl diphosphate synthase from *Abies grandis* and a 1,8-cineole synthase from *Hypoxilon sp*.	1.4 g/L	[53]
1,8-Cineole	1,8-Cineole synthase/ *streptomyces clavuligerus*	*Escherichia coli*	Using a 2-plasmid system with one plasmid having 1,8-cineole synthase and geranyl diphosphate synthase and another plasmid carrying mevalonate pathway genes	505 mg/L	[59]
(+)-Borneol	(+)-Bornyl diphosphate synthase/*Cinnamomum burmanni*	*Saccharomyces cerevisiae*	Introducing an N-terminal truncated (+)-bornyl diphosphate synthase alongside mevalonate pathway genes, and adding the Kozak sequence	2.89 mg/L	[54]
(−)-Borneol	(−)-Bornyl diphosphate synthase/*Blumea balsamifera*	*Saccharomyces cerevisiae*	Introducing an N-terminal truncated (−)-bornyl diphosphate synthase, adding the Kozak sequence, and using a double-mutated farnesyl diphosphate synthase. Using 5L bioreactor	148.59 mg/L	[55]
(−)-Borneol	Bornyl diphosphate synthase/ *Lippia dulcis*	*Escherichia coli*	Introducing a bornyl diphosphate synthase with a single-point mutation and more efficiency, and an endogenous phosphatase. Optimizing the fermentation	87.2 mg/L	[61]

##### Production of lavender monoterpenes in yeast

Deng et al. [48] produced linalool in *Saccharomyces cerevisiae* by introducing a fusion protein composed of LINS from *Actinidia arguta* and farnesyl diphosphate synthase [ERG20] from *S. cerevisiae*. The fusion protein, connected by a proper polypeptide linker between enzymes, exhibited a 69.7% increase in efficiency in linalool production compared to the application of individual free enzymes. Furthermore, Zhang et al. [49] demonstrated an enhancement in linalool production in *S. cerevisiae* through a series of experiments: They initially integrated the Isopentenol Utilization Pathway [IUP] into *S. cerevisiae* by incorporating choline kinase and isopentenyl phosphate kinase. Subsequently, LINS from *Actinidia arguta* was truncated from the N-terminal and introduced into *S. cerevisiae*. A double mutation was applied to ERG20 to enhance its efficiency, followed by its introduction into *S. cerevisiae*. linalool production was further enhanced by optimizing isoprenol, prenol, carbon source, and including Mg^2+^ in the medium. Moreover, Zhou et al. [50] applied a combinatorial strategy to enhance linalool content in *S. cerevisiae*. This strategy involved the overexpression of the entire MVA pathway, as well as a LINS from *Mentha citrata*, resulting in a significant increase in linalool concentration. Later, they further enhanced linalool production by employing a double mutation in LINS and ERG20. Zhang et al. [51] conducted dual metabolic engineering of the MVA pathway to upgrade linalool content in both the mitochondria and cytoplasm of *S. cerevisiae*. This was achieved by introducing MVA genes into both cellular compartments, with mitochondrial localization signal [MLS] fused to the genes for transfer into mitochondria. Thus, they constructed a strain of *S. cerevisiae* in which the expression of LINS from *Cinnamomum osmophloeum* and ERG20^F96W−N127W^ occurred in the cytoplasm and mitochondria. This recombinant *S. cerevisiae* exhibited increased linalool production (7.61 mg L^−1^). Moreover, they cultured the recombinant *S. cerevisiae* in media containing varying amounts of PYR and mevalonolactone as carbon sources. Notably, the medium supplemented with 70 mg L^−1^ mevalonolactone yielded the highest linalool concentration. Park et al. [52] enhanced linalool production by integrating an inducible sensor array into the genomic DNA of *S. cerevisiae*. This sensor array was comprised of sequences encoding repressor proteins controlled by constitutive promoters and strong terminators, allowing for individual or simultaneous expression upon exposure to sensor molecules in the medium. The sensor molecules employed included xylose, anhydrotetracycline, vanillic acid, and IPTG which induced the expression of LINS from *M. citrata*.

Kirby et al. [53] significantly enhanced 1,8-cineole production in *Rhodotorula toruloides* by employing an N-terminal truncated GPPS from *Abies grandis* and a CINS from *Hypoxilon sp.* These synthases were introduced into *R. toruloides* under the control of promoters sourced from the *R. toruloides* genome. Subsequently, the titer increased further through medium optimization. Ma et al. [54] overexpressed (+)-bornyl diphosphate synthase [BPPS] from *Cinnamomum burmanni* along with all genes involved in the MVA pathway in *S. cerevisiae* to produce (+)-borneol*.* (+)-Borneol production was further significantly increased by N-terminal truncation of BPPS and incorporating a Kozak sequence. In another study by the same researcher, Ma et al. [55], (−)-BPPS from *Blumea balsamifera* was identified and functionally characterized and then introduced into *S. cerevisiae*. Similar to the previous experiment, N-terminal truncation of BPPS and the addition of a Kozak sequence were utilized to enhance (−)-borneol production. Finally, the fusion of (−)-BPPS with ERG20^F96W−N127W^ resulted in a further increase in the production of (−)-borneol.

##### Production of lavender monoterpenes in *bacteria*

Wu et al. [56] employed a scaffolding strategy to produce linalool in *E. coli*. They constructed scaffolds consisting of three domains for IPPS, GPPS and LINS enzymes, with different domain repeats for GPPS and LINS. Ligands were attached to the enzymes via linkers. The scaffold featuring one domain for IPPS, one for GPPS, and four for LINS exhibited the highest linalool production. Additionally, they optimized the concentrations of IPTG (0.5 mM), L-arabinose (0.3%), and glycerol (4%) in the medium, with an identified optimal temperature of 20º C for linalool production. Kong et al. [57] produced linalool in *E. coli* by designing and introducing a heterologous MVA pathway, which included genes involved in IPP and DMAPP accumulation, GPP formation, and linalool production. They showed that GPPS2 from *A. grandis* had a more significant effect on linalool production compared to ERG20. The lower efficiency of ERG20 in linalool production was attributed to its bifunctional activity, resulting in the production of both GPP and FPP. The recombinant *E. coli* strain harboring the new MVA pathway produced 15 ± 1.4 mg L^−1^ linalool. The linalool concentration further increased to 63 ± 5.6 mg L^−1^ with the overexpression of isopentenyl diphosphate isomerases. In a study by Wang et al. [58], linalool production in *E. coli* was enhanced through LINS modification. Initially, the most efficient LINS (4.8 mg L^−1^) was obtained from *Streptomyces clavuligerus* [bLIS]. Then, bLIS variants with different ribosomal binding sites [RBS] and translation initiation rate [TIR] were constructed. The results demonstrated a positive correlation between bLIS expression and TIR. Additionally, a fusion tag was added to increase bLIS solubility, resulting in enhanced linalool production to 33.4 mg L^−1^. Further optimization strategy included the addition of GPSS from *A. grandis* to ensure sufficient GPP availability, leading to linalool production reaching 100.1 mg L^−1^. Finally, culturing the recombinant *E. coli* in a bioreactor with fed-batch fermentation achieved a significant increase in linalool production to 1027.3 mg L^−1^.

Mendez-Perez et al. [59] achieved significant 1,8-cineole production (228 mg L^−1^) in *E. coli* by constructing and introducing a plasmid harboring genes related to the MVA pathway along with the CINS gene from *Streptomyces clavuligerus*. To further enhance 1,8-cineole production, they inserted an additional plasmid containing another copy of CINS into *E. coli*. This two-plasmid system led to a 33% increase in 1,8-cineole production, reaching up to 305 mg L^−1^. The higher level of 1,8-cineole (505 mg L^−1^) was achieved when CINS and GPPS were harbored in one plasmid and other genes were placed in another plasmid. Karuppiah et al. [60] inserted LINS and CINS genes from *Streptomyces clavuligerus* into an engineered *E. coli* strain, where the MVA pathway was regulated by an IPTG-inducible promoter, and an N-terminal truncated GPPS was controlled by a tetracycline-inducible promoter. This approach resulted in the production of a remarkable amount of linalool and 1,8-cineole, with the latter exhibiting a higher purity (96%) compared to those obtained from *Salvia fruticose*, *Arabidopsis thaliana* and *Citrus unshiu* which had purities of 67%, 42% and 63%, respectively.

Lei et al. [61] engineered the de novo production of borneol in *E. coli*. They co-expressed a mutant BPPS enzyme from *Lippia dulcis*, where a single-point mutation enhanced enzymatic activity, along with an endogenous phosphatase from *E. coli* to facilitate the dephosphorylation of precursors to borneol. This strategy led to a notable enhancement in borneol content under optimized fermentation condition.

##### Production of lavender monoterpenes in Cyanobacteria

Matsudaira et al. [62] engineered a cyanobacterium strain capable of producing S-linalool. In this strain, the LINS coding sequence from *Actinidia arguta* was codon-optimized for the cyanobacterium and expressed under the control of *tac* promoter. This strain produced 11.4 mg L^−1^ of S-linalool in shake flask culture. The S-linalool concentration further increased to 11.6 mg L^−1^ with the expression of a mutated farnesyl diphosphate synthase derived from E. coli.

Sakamaki et al. [63] reported the photosynthetic production of 1,8-cineole in cyanobacteria. They designed and constructed a codon-optimized CINS gene from *Streptomyces clavuligerus* for producing 1,8-cineole in *Synechococcus elongatus.* They placed this CINS under the control of an IPTG-dependent promoter since it could produce 1,8-cineole directly from GPP, unlike other CINS that convert terpineol into 1,8-cineole. Although the amount of 1,8-cineole produced in their attempt was not remarkable and further attempts are needed, their study showed the feasibility of producing 1,8-cineole without the need for carbon sources like sucrose.

## Conclusion

By now, most terpene synthase genes responsible for the production of lavender EO monoterpenes have been cloned and functionally characterized. Further, many of these genes have been used to produce the corresponding monoterpenes in bacteria, yeast, and model plants such as Arabidopsis and tobacco. Additionally, the metabolic engineering of lavender has been investigated. In the latter case, success has been limited as the constitutive overexpression of terpene synthase genes is often detrimental to the host plant, as (presumably) non-GT cells cannot tolerate large amounts of the monoterpenes they produce. This problem may be resolved if GT-specific promoters that can direct the expression of transgenes specifically in GTs are used. In this context, ongoing studies currently focus on the cloning of GT-specific promoters. Such promoters could not only help enhance EO quality and yield in lavender, but also assist researchers in using this plant as a bioreactor for the large-scale production of valuable phytochemicals.

## Data Availability

Not applicable.

## Abbreviations

AT: Acetyltransferase
BDH: (+)-Borneol dehydrogenase
bHLH: Basic helix-loop-helix
bLIS: LINS (from Streptomyces clavuligerus)
BPP: Bornyl diphosphate
BPPS: Bornyl diphosphate synthase
CaMV: Cauliflower Mosaic Virus
CINS: 1,8-Cineole synthase
CMK: 4-(Cytidine 5′-diphospho)- 2-C-methyl-D-erythritol-kinase
DMAPP: Dimethylallyl diphosphate
DXP: 1-Deoxy-D-xylulose-5-phosphate
DXR: DXP reductoisomerase
DXS: DXP synthase
EOs: Essential oils
ERG20: Farnesyl diphosphate synthase
FPP: Farnesyl diphosphate
G3P: Glyceraldehyde 3-phosphate
GC–MS: Gas chromatography/mass spectrometry
GGPP: Geranylgeranyl diphosphate
GPP: Geranyl diphosphate
GPPS: Geranyl diphosphate synthase
GT: Glandular trichome
HDS: HMBPP synthase
HDR: HMBPP reductase
HMBPP: 1-Hydroxy-2-methyl-2-(E)-butenyl-4-diphosphate
IDSs: Isoprenyl diphosphate synthases
IPP: Isopentenyl diphosphate
IUP: Isopentenol Utilization Pathway
LaAG-like: LaAGAMOUS-like (from L. angustifolia)
LaBERS: Bergamotene synthase (from L. angustifolia)
LabPHLS: β-Phellandrene synthase (from L. angustifolia)
LaSEP3-like: LaSEPALLATA3- like (from L. angustifolia)
LaSVP: Short vegetative phase gene (from l. angustifolia)
LaTPS7: Terpene synthase 7 (from L. angustifolia)
LaTPS8: Terpene synthase 7 (from L. angustifolia)
Li3CARS: 3-Carene synthase (from L. x intermedia)
LiAAT: Alcohol acetyltransferase (from L. x intermedia)
LiBDH: Borneol dehydrogenase (from L. x intermedia)
LiBPPS: Bornyl diphosphate synthase (from L. x intermedia)
LiCINS: CINS (from L. x intermedia)
LiCPS: Caryophyllene synthase (from L. x intermedia)
LiLPPS: Lavandulyl diphosphate synthase (from L. x intermedia)
LIMS: Limonene synthase
LINS: Linalool synthase
LiS-LINS: S-linalool synthase (from L. x intermedia)
LpFENS: Fenchol synthase (from L. pedunculata)
LpGEAS: Germacrene A synthase (from L. pedunculata)
LpPINS: α-Pinene synthase (from L. pedunculata)
LPP: Lavandulyl diphosphate
LPPS: Lavandulyl diphosphate synthase
MCT: MEP cytidyltransferase
MDS: MEcPP synthase
MEcPP: 2-C-methyl-D-erythritol -2,4-cyclodiphosphate
MeJA: Methyl jasmonate
MEP: 2-C-Methyl-D-erythritol 4-phosphate
MLS: Mitochondrial localization signal
monoTPSs: Monoterpene synthases
MVA: Mevalonate
NPP: Neryl diphosphate
NPPS: Neryl diphosphate synthase
PYR: Pyruvate
RBS: Ribosomal binding site
SesquiTPSs: Sesquiterpene synthases
TFs: Transcription factors
TIR: Translation initiation rates
TPS: Terpene synthase
VOC: Volatile organic compound
